# Effect of Ramadan Fasting on Body Weight and Body Mass Index (BMI) in Public Sector Undergraduate Medical Students of Peshawar

**DOI:** 10.12669/pjms.39.3.7017

**Published:** 2023

**Authors:** Abdul Majid, Muhammad Osama, Muhammad Noman, Ulfat Nisa, Iqbal Haider

**Affiliations:** 1 Abdul Majid, Final Year MBBS Khyber Medical College Peshawar, Peshawar, Pakistan; 2 Muhammad Osama, Final Year MBBS Khyber Medical College Peshawar, Peshawar, Pakistan; 3 Muhammad Noman, Final Year MBBS Khyber Medical College Peshawar, Peshawar, Pakistan; 4 Ulfat Nisa, Final Year MBBS Khyber Medical College Peshawar, Peshawar, Pakistan; 5 Dr. Iqbal Haider, Department of Medicine, Khyber Medical College Peshawar, Peshawar, Pakistan

**Keywords:** Weight, BMI, Fasting, Medical Students

## Abstract

**Objective:**

To determine the effects of fasting on weight and eventually on Body Mass Index (BMI) in medical students of a Public Sector Medical College

**Methods:**

It was a prospective analytical study conducted in a Public Sector Medical College in Peshawar City from 28^th^ March to 20^th^ May 2022 (1443 Hijri). Convenience Sampling was used and 115 students (58 male and 57 female) of 1^st^ Year MBBS to Final Year MBBS were enrolled. Four readings of weight were taken, one before, two during, and one after Ramadan. A well-structured self-administered questionnaire was used to inquire about basic demographic characteristics, sleep patterns during Ramadan and normal routine, and family history of obesity. The collected data were analyzed through SPSS software and a repeated measures ANOVA test was used for drawing statistical conclusions.

**Results:**

A slight increase in the mean weight was observed during the second week of Ramadan while a loss of 0.4 kg occurred during the fourth week of Ramadan, F (1, 81) = 1777.55; p < 0.0001. The same pattern was observed for BMI, F (1, 81) = 2705.18; p < 0.0001. However, the weight and the BMI were regained in two to three weeks following Ramadan.

**Conclusion:**

Ramadan offers a non-hazardous way of weight loss. Further studies across different geographical locations with larger sample sizes should be conducted to identify and quantify the association between weight and fasting and also to identify potential confounders.

## INTRODUCTION

Worldwide 2.1 billion people are considered overweight or obese and its prevalence is increasing like an epidemic; obesity is linked to some serious health conditions including, type-2 diabetes, hypertension, coronary heart disease, stroke, gallbladder disease, respiratory problems, sleep apnea, osteoarthritis, psychological consequences including depression, impaired body image, and low self-esteem and even cancer, the worst scourge of civilized mankind.^1^,^2^

There are many ways to lose weight, including exercise, fasting, special diets, the use of drugs, hormonal therapy, and so on. Intermittent fasting can be a very efficient way of losing weight. Three intermittent fasting regimens have been studied in the scientific literature in much detail: alternate-day fasting, fasting two days a week and time-restricted feeding all have been shown to be beneficial.^3^ Many studies have shown that it is primarily the weight loss that accompanies intermittent fasting that leads to getting benefits and reducing the risks of the above-mentioned conditions.^4^ Fasting has been well ingrained in the Islamic religion with Ramadan fasting being obligatory worship while other practices such as fasting three days a month (on the 13^th^, 14^th^ and 15^th^) or two days a week (on Mondays and Thursdays) are optional but recommended and preferable.^5^

Ramadan is the holiest month of the Islamic religion in which millions of Muslims fast and abstain from eating and drinking from sunrise to sunset. The beneficial effects of fasting on the human body are well known today, with studies reporting weight loss, increase in life span, decreased oxidative stress, and improvement in inflammatory markers with decreased levels of Interleukin-6 (IL-6) and Tumor necrosis Factor-α (TNF-α), both of which are related to adverse health conditions, for example, some cardiovascular and psychiatric diseases.^6^,^7^ Ramadan fasting leads to a decrease in TNF-α and an increase in adiponectin levels (decreased adiponectin is associated with obesity, diabetes, and atherosclerosis).^8^

Although intermittent fasting has been known to lead to weight loss, studies on the effect of fasting in Ramadan on weight have shown conflicting results with some studies showing no changes in weight or weight gain while others show weight loss.^9^,^10^ According to our observation, the dietary patterns, levels of physical activity, preference for fatty and sugary food, and sleep patterns of Pakistani people during Ramadan are significantly different from other countries due to which a weight gain, not a loss may occur during the month of Ramadan. This pattern has also been observed in Saudi Arabia.^10^ Further, very few if any studies could be found that address this problem in our country, we could find only one study that assessed changes in weight during Ramadan but that was conducted on diabetic patients.^11^ Thus, we are undertaking this endeavour to determine the effect of fasting on weight and eventually BMI (Body mass index). We’ll also be taking into account various factors that may affect our study such as sleep patterns and family history of obesity.

## METHODS

It was a Prospective analytic study conducted in a Public Sector Medical College in Peshawar City. This study was conducted between 28^th^ March to 20^th^ May 2022 (1443 Hijri). Convenience Sampling was used and 115 students (58 male and 57 female) of 1^st^ Year MBBS to Final Year MBBS were enrolled. The sample size calculation was based on a previous study conducted in Saudi Arabia.^10^ Participants included those who provided consent to be included in our study, were fasting in Ramadan, were looking physically healthy, and had no underlying chronic disease to the best of their knowledge. Excluded participants were those that didn’t provide consent, Students who were not fasting or had missed more than seven fasts in Ramadan, were using any medicine that had effects on weight, and unhealthy, diseased students. Table-I

**Table-I:** Demographic characteristics of participants

No. of Students	Age (Mean ± SD)	Gender		Year of Study (MBBS)	Family History of Obesity		Sleep Hours During Normal Routine (Mean ± SD)	Sleep Hours During Ramadan (Mean ± SD)
		*Male*	*Female*	1st Year: 21	Yes	No		
				2nd Year: 23				
115	21.25 ± 1.896	58	57	3rd Year: 22	31	84	7.61 ± 1.08	7.46 ± 1.86
				4th Year: 23				
				5th Year: 26				

Ethical approval was obtained from The Ethical Board of Khyber Medical College Peshawar (382/DME/KMC; dated 21-3-2022). Four readings of weight were taken, the first one three to seven days before Ramadan (pre-Ramadan), the second during the second week of Ramadan, the third in the fourth week of Ramadan, and the final fourth reading two to three weeks after Ramadan (post-Ramadan). Digital weight balance (Beurer PS 240, made in Germany) was utilized for this purpose. A measuring tape was used for recording height as it was needed for calculating BMI. A well-structured self-administered questionnaire was used to inquire about basic demographic characteristics, sleep patterns during Ramadan and normal routine, and family history of obesity. The Data were collected physically by measuring the weight and height of the students included in the study sample. The collected data were analyzed through SPSS software (IBM Corp. Released 2019. IBM SPSS Statistics for Windows, Version 26.0. Armonk, NY: IBM Corp.) Repeated measures ANOVA test was used for drawing statistical conclusions.

## RESULTS

**Fig.1:**
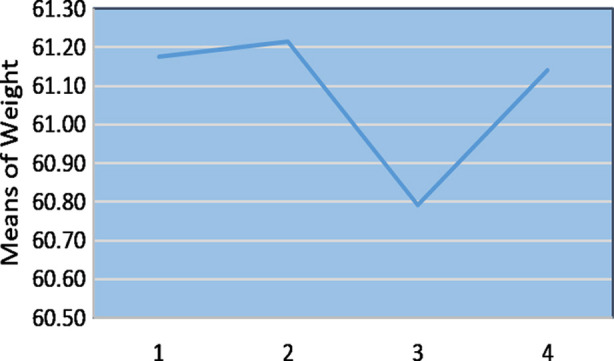
Graphical Representation of Weight Changes 1. Pre-Ramadan 2. Second Week of Ramadan 3. Fourth Week of Ramadan 4. Post-Ramadan

**Fig.2:**
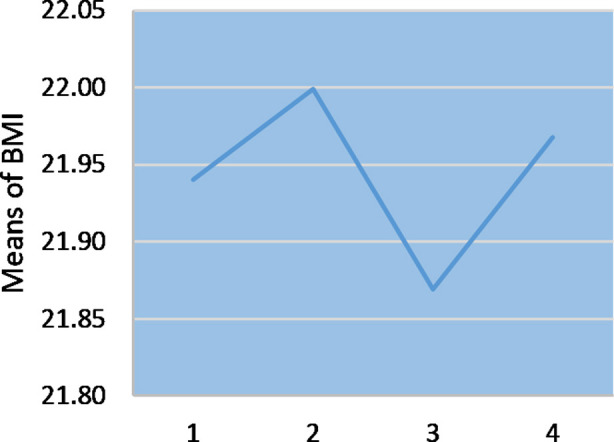
Graphical Representation of BMI Changes 1. Pre-Ramadan 2. Second Week of Ramadan 3. Fourth Week of Ramadan 4. Post-Ramadan

Out of 115 participants enrolled, two participants in the second reading, 28 participants in the third reading, and four participants in the final reading (post-Ramadan) were lost to follow-up, analysis of the remaining showed a statistically significant association between fasting during Ramadan and weight as was evident from the interpretation of Repeated measures ANOVA test F (1, 81) = 1777.55; p < 0.0001. Significant weight changes had occurred during the 2^nd^ and 4^th^ week of Ramadan (p = 0.001) as was illustrated by pairwise comparison of the groups. A mean weight loss of 0.4 kg occurred during the fourth week of Ramadan, paradoxically a slight increase in the mean weight was observed during the second week of Ramadan (Table-II; Fig.1). A significant change in BMI, F (1, 81) = 2705.18; p < 0.0001 was also observed with the same pattern i.e., increase till the second week and then decrease was observed in the last week of Ramadan (Fig.2; Table-III). A pairwise comparison showed that significant changes in BMI had occurred between the second and fourth week of Ramadan (p = 0.038). However, the weight and BMI were regained in two to three weeks following Ramadan (Table-II; Table-III).

**Table-II:** Weight changes

Intervals	Weight in kg (Mean ± SD)	95% Confidence Interval	

		Lower Limit	Upper Limit
Pre-Ramadan	61.174 ± 13.117	58.292	64.057
2^nd^ week	61.214 ± 13.104	58.334	64.093
4^th^ week	60.792 ± 13.178	57.896	63.688
Post-Ramadan	61.142 ± 13.309	58.217	64.066

**Table-III:** Changes in BMI

Intervals	BMI (Mean ± SD)	95% Confidence Interval	

		Lower Limit	Upper Limit
Pre-Ramadan	21.9404 ± 3.81	21.103	22.778
2^nd^ week	21.9988 ± 3.84	21.155	22.843
4^th^ week	21.8691 ± 3.85	21.022	22.717
Post-Ramadan	21.9678 ± 3.88	21.113	22.822

For both weight and BMI, Mauchly’s Test of Sphericity was applied with a p-value < 0.0001 thus the assumption of sphericity is violated and we can say the variances between the groups are not the same. Pillai’s Trace and Wilks’ Lambda tests also show significant differences between the groups (p = 0.002 for weight and p = 0.048 for BMI).

## DISCUSSION

A statistically significant association was found between weight and fasting during the month of Ramadan. A slight increase in weight was observed in the second week of Ramadan followed by a decline in the last week. However, the weight was regained in about two to three weeks after Ramadan. Various studies conducted across different geographical locations have reported conflicting findings about weight and fasting in the month of Ramadan. A study conducted in Saudi Arabia reported a weight gain during Ramadan while another study reported that about one kg of weight loss occurs but that weight was quickly regained shortly after Ramadan.^10^,^12^ A systematic review and meta-analysis also reported similar findings with a mean weight loss of 1.51 kg for men and 0.92 kg for women.^13^ Another review shows significant weight loss in males but no significant changes in females.^14^ Nonetheless, another systematic review found a weight loss of 1.34 kg but also states that greater weight loss occurred in those participants who had a higher pre-Ramadan BMI.^15^ As in our study, the majority of the studies reveal that this weight is regained two to three weeks after Ramadan.^12^

A few studies have also reported a weight gain or no change in weight but the majority are in favour that generally, weight loss occurs during Ramadan.^10^,^16^ Although certain confounding factors may be responsible for this discrepancy such as the duration of fasts (as it varies geographically), cigarette smoking, obesity, dietary patterns, medications, and cultural habits, etc.^17^

We suggest that if fasting is observed in the coming months after Ramadan, it may lead to a sustained decrease in weight as is shown by a study that intermittent fasting for six months leads to a decrease in BMI, weight, and body fat mass.^18^ As both Ramadan and intermittent fasting leads to weight loss, physicians can advise the various intermittent fasting regimens (whatever is suitable for the patient) after Ramadan and can achieve significant weight loss and its added benefits. Various studies have shown that intermittent fasting reduces insulin resistance and improves cardiometabolic profile in diabetic and obese patients respectively.^19^ In patients with cardiovascular diseases, an improvement in blood pressure, heart rate, high-density lipoproteins (HDL) and low-density lipoproteins (LDL) have been observed.^20^ Various animal studies and some clinical trials have demonstrated decreased tumor growth, increased survival and a better response to therapy.^21^ Although the benefits of fasting are far-reaching, incorporating it into patients’ lives remains a major challenge for healthcare personnel.

Our study has shown a light of hope in what could turn out to be a way of getting rid of excess body weight instead of exposing oneself to harmful medications and other much riskier means of weight loss. It just needs a little effort and a will to a healthy life; may it be in the form of fasting in Muslims or restricted feeding times or any intermittent fasting regimens in other sects. Further studies across different geographical locations should be arranged to correctly identify and quantify this association and also to identify potential confounders.

### Limitations:

As we were constrained by lack of resources and time, we were limited to a small sample size and included only MBBS students that are considered to have a comparatively more sedentary lifestyle. The study would have been more fruitful if we had included students of other specialties/departments that were difficult and seemingly impossible for us owing to the limitation of resources and time. But as other studies show the same effect in the general population as well, the result is expected to be the same if observed in any subset of the population.

## CONCLUSION

Ramadan fasting offers a benign non-hazardous way of weight loss, especially for the Muslim population. It can also help in maintaining a strict diet after Ramadan. Further studies across different geographical locations with larger sample sizes should be arranged to precisely identify and quantify the association between weight and fasting and also to identify potential confounders.
